# The Book World of Medicine and Science

**Published:** 1899-11-25

**Authors:** 


### The Book World of Medicine and Science.

Urinary Analysis and Diagnosis. By Louis Heitzmann, M.D.New York. (London: Baillière, Tindall, and Cox. 1899. Pp. 253. Illustrated. Price 10s. 6d.)

Dr. Heitzmann, in his prefatory apology for adding yet another to the long list of works on urinary analysis, admits the brevity of his remarks on chemical diagnosis, and justifies his authorship by a claim to the more exhaustive consideration of microscopical diagnosis.

We should be inclined to call the first portion of this work —that devoted to chemical analysis—quite inadequate. Indeed, it might with advantage have been altogether omitted, since ampler information may be found in any good text-book of medicine. To the second and third sections, however—those on microscopical examination and diagnosis—Dr. Heitzmann compels our attention. We are bound to say we have been surprised at the cursory dismissal of many important points. In the section devoted to the gonococcus, however, technique is amply discussed, but the pseudo-tubercle bacillus of urine—the smegma bacillus—is ignored, and the typhoid and colon bacilli are somewhat inadequately treated of. The author attempts to differentiate microscopically varieties of vaginitis and cystitis—surely quite hopelessly. The diagrams are ambitious and original, but they all suffer from being on too large a scale. There is no object—and indeed it is misleading—to illustrate spermato-cystitis by a full-page drawing in which each spermatozoon is three-quarters of an inch long. So, too, in representing casts two and three inches long nothing is gained but a magnification of errors in draughtsmanship. We suppose it is hopeless to expect our Transatlantic brethren to spell fibre and centre anyhow but "fiber" and "center," but this is a detail.

The Law Relating to Infectious Diseases and Hospitals. By H. H. Copnall, Solicitor, Deputy Clerk and Clerk to the Committees of the Wilts County Council. (London: Hadden, Best, and Co. 1899. Price 7s. 6d. net.)

This is a book which will be found of the greatest possible service to all who have to do with putting in force the law in regard to infectious diseases, and the establishment and management of hospitals for their treatment and control. Broadly, no doubt, it may be said that the law on the subject is to be found in a few Acts of Parliament, but when we come to details it will be found that the number of Acts and General Orders, and of official memoranda giving directions as to the working of their provisions, is very considerable; and there can be no doubt that the author of this book has done a most useful piece of work in collecting together under one cover the sections of these various enactments bearing on the subject. The work is divided into three parts and an introduction. The introduction gives a general outline of the form that legislation on the subject has taken, and of the course which has to be adopted by the various bodies concerned in the control of infectious disease. Then comes in Part I., a series of extracts from Acts of Parliament, &c., fully annotated and with plenty of cross references; in Part II. we have extracts from various memoranda, such as that by the Medical Officer of the Local Government Board on "The Steps Requisite to be Taken Where Small-pox is Prevalent," on "The Provision of Isolation Hospital Accommodation," on "Influenza," on "The Closure of Public Schools," &c.; and, finally, in Part III., there is a short digest of decided cases. Altogether this will be found a most useful and time-saving book, giving as it does the exact wording of the sections and paragraphs dealing with the subject, always a most important matter. It is not a mere key to documents one may not possess, but gives the necessary quotations.

Architectural Hygiene; or, Sanitary Science as Applied to Buildings. By Banister F. Fletcher and H. Phillips Fletcher. (London: D. Fourdrinier. 1899. Price 5s.)

This, which is one of "'The Builder' Student's Series," is but a small book, but it is wonderfully complete considering its size. Its aim is to serve as "a concise yet complete text book, treating the subject in all its branches so far as it affects architects, surveyors, engineers, medical officers of health, sanitary inspectors, plumbers, and students generally," and when we find it stated that "the subject is treated *ab initio*, *i.e.*, from the foundation of a building to its finishing and furnishing," we need hardly say that the authors have set themselves a somewhat mighty task. If to this we add that the product of their labours is contained in 268 small pages, it will be seen that from the necessities of the case many of the subjects dealt with have had to be treated in a short and not entirely exhaustive manner. Still we know of no book on the subject which covers so large a field and contains so concentrated a mass of information, and we can fully recommend it to the student as likely to be of very great assistance to him in mastering the elements of architectural hygiene. We doubt, however, whether the medical officer of health will find in it much with which he is unacquainted, but it is a book to which those studying for that post may well resort. The chapters on heating and ventilation will, we hope, be found very useful by the rising generation of architectural students, for many points are insisted upon by the writers which architects of the present day, and even architects of high position, seem as yet to have failed to apprehend. "We cannot condemn too strongly," say the authors, "the placing of hot-water pipes below a floor level, covered by gratings; such ducts become the receptacles of all sorts of filth, which dries into dust and is carried about by the air current set up, and contaminates the atmosphere to be breathed." Yet how common these abominations are! Only a few nights ago we had the delight of sitting over one of these "receptacles for all sorts of filth," and fully experiencing how this filth "contaminates the atmosphere," at one of the best known of the London concert halls—a building that has not been erected more than about half a dozen years. So that the caution given above is by no means superfluous. Turning to the chapter on house ventilation and warming schemes, we would quote the following, with which we thoroughly agree: "The great secret of a warmed and ventilated house lies in thoroughly attending to the hall and staircase, and no system will be found complete until this is taken in hand." If the air in the hall is kept within a trifle as warm as that in the sitting-rooms the problem of ventilating the latter without perceptible draught is reduced to its simplest proportions. The chapters on the making of sanitary inspections and on drawing up reports are very good, and the illustrative cases will be found useful as types. The book is well illustrated by a large number of woodcuts, which, although in some instances rather minute, are so clearly printed as to serve their purpose very well.

### BOOKS RECEIVED.

Simpkin, Marshall, and Co.

"The Maintenance of Health in Egypt." By Arthur J. M. Bentley, M.D.

J. and A. Churchill.

"The Pathologist's Handbook: A Manual for the Post-mortem Room." By T. N. Kelynack, M.D., M.R.C.P.

D. Fourdrinier, "Builder" Office.

"Architectural Hygiene; or, Sanitary Science as Applied to Buildings." By Banister F. Fletcher and H. Phillips Fletcher.

